# Identification of Quantitative Trait Loci Controlling High Calcium Response in *Arabidopsis thaliana*

**DOI:** 10.1371/journal.pone.0112511

**Published:** 2014-11-17

**Authors:** Wenlong Li, Huikun Duan, Fengying Chen, Zhi Wang, Xueqing Huang, Xin Deng, Yongxiu Liu

**Affiliations:** 1 Key Laboratory of Plant Resources, Institute of Botany, Chinese Academy of Sciences, Beijing 100093, China; 2 Key Laboratory of Plant Molecular Physiology, Institute of Botany, Chinese Academy of Sciences, Beijing 100093, China; 3 State Key Laboratory of Genetic Engineering, Institute of Plant Biology, School of Life Sciences, Fudan University, Shanghai 200433, China; 4 College of Life Sciences, University of Chinese Academy of Sciences, Beijing 100049, China; Nanjing Forestry University, China

## Abstract

Natural variation for primary root growth response to high Ca stress in *Arabidopsis thaliana* was studied by screening a series of accessions (ecotypes) under high Calcium (40 mM CaCl_2_ ) conditions. The genetic basis of this variation was further investigated by QTL analysis using recombinant inbred lines from Landsberg *erecta* (L*er*)×Cape Verde Islands (Cvi) cross. Four QTLs were identified in chromosome 1, 2 and 5,and named response to high Calcium (*RHCA*) 1–4. The three QTLs (*RHCA1*, *RHCA2* and *RHCA4*) were further confirmed by analysis of near isogenic lines harboring Cvi introgression fragments in L*er* background. Real-time PCR analysis showed that several genes associated with high Ca response including *SMT1* and *XHT25* have changed expression pattern between L*er* and near isogenic lines. These results were useful for detecting molecular mechanisms of plants for high Ca adaption.

## Introduction

Ca is an essential plant macronutrient with key structural and signaling roles. Ca ions (Ca^2+^) act as an osmoticum within vacuoles, a stabilizing element in the membranes, a strengthening agent in cell walls, and a secondary messenger for a multitude of signals [1]–[3]. Keeping Ca content appropriately in plants is essential for their normal growth. Local Ca deficiency affects the development of the cell wall and causes local cell necrosis [4], leads to symptoms in organs with low transpiration such as the tipburn in young expanding leaves, black heart of celery in enclosed tissues and bitter pit of apples [5]. However, high Ca in the rhizospheric solution may also cause Ca toxicity, such as preventing the germination of seeds, reducing plant growth rates and forming tiny yellowish or gold spots in the cell walls of fruits [6]–[8].

In spacious karst areas, the toxification of excessive Ca to plants significantly inhibits plant growth, crop production and species distribution [3], [9]–[11]. Calcium is very rich in the soil of karst regions, nearly three times more than in acid soils [10], [12]. The exchangeable Ca contents in plant rhizospheric soil from typical karst area in Guizhou province of China were nearly 1500 mg/kg (data unpublished), which was far more than that in loamy sand (300 mg/kg), silt loam (650 mg/kg) and sand loam (700 mg/kg) [13], [14]. Karst terrain accounts for about 15% of the world’s land area, about 2.2 million km^2^, and is home for around 1 billion people (17% of the world’s population) [15]. Importantly, biodiversity and agricultural production in the vast area of karst regions, particularly in Europe, Asia, North and Central America and Caribbean [16], have been threatened by the high Ca content in the soil. Generating crops and economically important plants with tolerance to high calcium (internal or external) will help to develop the sustainable agriculture, ecosystem and economics in these areas. From this point of view, exploring molecular mechanisms of plants for high Ca response is highly desired.

Several studies have focused on the molecular evens under high Ca condition. Genome-wide analysis displayed that 420 and 199 transcripts were found to be up- and down-regulated under high Ca stress in *Amaranthus hypochondriacus* respectively, many of the genes are associated with stress response, transcription regulation and signal transduction [17]. In *Arabidopsis*, the mutants of *sterol methyltransferase 1* (*SMT1*) were hypersensitive to to higher Ca concentrations [18]. *SMT1* could enhance the resistance of plants to high Ca stress. Cyclic nucleotide-gated channel 2 (CNGC2) was identified to play a key role in adaptation to high external Ca condition, the *cngc2* mutant showed clearly increased sensitivity to external high calcium, and its transcription profile grown in normal media resembled that from wild type plants grown under elevated exogenous calcium condition [7], [19]. CAX3, a calcium antiporter, is induced by exogenous calcium, and *cax3* mutants are also marginally sensitive to high external calcium [7], [19]. *Brassica juncea* PCR1 (*BjPCR1*), a member of the plant cadmium resistance (PCR) protein family in Indian mustard, is an exporter required for the translocation of Ca^2+^ from the root epidermis to the inner cells and ultimately to the shoot. Root hair-specific expression of *BjPCR1* in *Arabidopsis* causes increased Ca^2+^ resistance and translocation [6]. The genetic dissection of high calcium stress response is still deficient and difficult; however, it is necessary for the development of high calcium adaptive cultivars.

QTL analysis has been employed as a powerful approach to discover molecular mechanisms of diverse plant traits [20]–[23]. However, so far, no reports on unraveling the genetic basis of high Ca stress response by QTL analysis were published. Thus, the objective of this research was to identify QTLs and possible candidate genes in *Arabidopsis* for high calcium stress response, towards the long-term goals of developing genetic tools that will facilitate the breeding of more high-calcium-adaptive cultivars.

## Materials and Methods

### Plant material

Accessions of *Arabidopsis thaliana* used were: Shakdara (Sha), Cape Verde Islands (Cvi), Landsberg *erecta* (L*er*), An-1(N99), Columbia (Col) and Eri. For QTL analysis, we used the Core-Pop set of 161 RIL lines for QTL mapping experiments [24]. L*er*/Cvi near-isogenic lines (NILs) were used for the confirmation and fine analysis of QTLs [25], [26].

### Plant growth and measurements

Seeds were treated with 70% ethanol for 30 seconds, and then surface sterilized with 0.7% sodium hypochlorite for 15–20 minutes. After washing 4 times with steriled water, the seeds were sowed on Murashige and Skoog (MS) salt (*Phyto*Technology Laboratories, Kansas, USA) agar (0.6%) medium supplemented with 1% sucrose. The Ca and Cl content in MS basic medium were about 3 mM and 6 mM respectively. After placed at 4°C for 5 days, the seeds were germinated at 23°C under illumination by light for 16 h and 8 h dark for 3 days. Subsequently, seedlings were transferred to MS agar (1%) medium supplemented with 1% sucrose with or without 40 and 50 mM CaCl_2_, 40 mM NaCl, 80 mM KCl and 40 mM Ca(NO_3_)_2_ respectively. Then, seedlings were grown vertically under photoperiodic cycles of 16 h of light and 8 h of dark at 23°C for 10 days. Finally, photographs were taken, and primary root lengths of all plantlets were measured. At least 3 seedlings per line per plate for each treatment were assayed. Three independent experiments with two or more replicates were conducted.

### QTL mapping and analysis

A total of 161 recombinant inbred lines (RILs) were derived from Landsberg erecta (L*er*) and Cape Verde Islands (Cvi). These lines were previously described and characterized using amplified fragment length polymorphism (AFLP) and cleaved amplified polymorphic sequence (CAPS) markers [24]. Meanwhile, primary root lengths of the parental lines and the 161 RIL lines were measured under control and high calcium conditions. QTL detections were performed by MapQTL (version 5.0, http://www.kyazma.nl) using both interval mapping (IM) and multiple-QTL model mapping (MQM) methods, as described in the MapQTL reference manual. A logarithm of the odds (LOD) threshold of 2.4 was applied to declare the presence of a QTL [27].

### Confirmation and fine analysis of QTLs

To validate the presence and the effect of QTLs, primary root lengths of 92 L*er*/Cvi near-isogenic lines (NILs) under control and high calcium condition were measured. High Ca sensitive NIL lines were obtained for further analysis. These NIL lines were also described and characterized using amplified fragment length polymorphism (AFLP) and cleaved amplified polymorphic sequence (CAPS) markers [25], [26].

### RNA isolation and Real-time PCR

Five-day-old seedlings were soaked in liquid MS medium with or without 40 mM CaCl_2_ for 24 hours. And then, total RNA was isolated using the trizol method with TRIzol Reagent (Takara, D9108B). After digestion with DNase I, it was reversely transcribed into cDNA with Oligo(dT)18 primer using SuperScript III reverse transcriptase, and used as template for PCR amplification. *Actin2* gene was used as internal references. Real-time PCRs were performed in a Mastercycler ep Realplex apparatus (Eppendorf, Hamburg, Germany) with SYBR Green Real-time PCR Master Mix (TOYOBO, Japan). The relative expression levels were determined using 2^−ΔΔCt^ method [28]. The expression level of each transcript was measured in three independent biological samples with three technical replicates. Real-time PCR forward and reverse primers of key genes were as follows: 5′-TGCACAAAGATGGAAAGGAG-3′ and 5′-CGGTAACAACTGAATTGCTG-3′ for *SMT1* (At5g13710); 5′-CATCTCCTCCTTCTAGCACCA-3′ and 5′-TAGGAAAGGAGCAGAGGGTCT-3′ for At1g21110; 5′-AGATCGGACCAGACCGTGTT-3′ and 5′-CGCTTTCCTCATCGGCATTA-3′ for At1g76680; 5′-CGCGTTCCATAGACTCGAGTA-3′ and 5′-ATGGAACCCTCATCACATCGT-3′ for At5g57550.

### Statistic analysis

All data were subjected to a one-way ANOVA analysis followed by DUNCAN (alpha = 0.05) test using statistical package SAS 9.2, and the results were displayed as means ± standard deviation. Each data was obtained from at least three independent samples.

## Results

### Natural variation of *Arabidopsis* in response to high calcium

**Figure 1:**
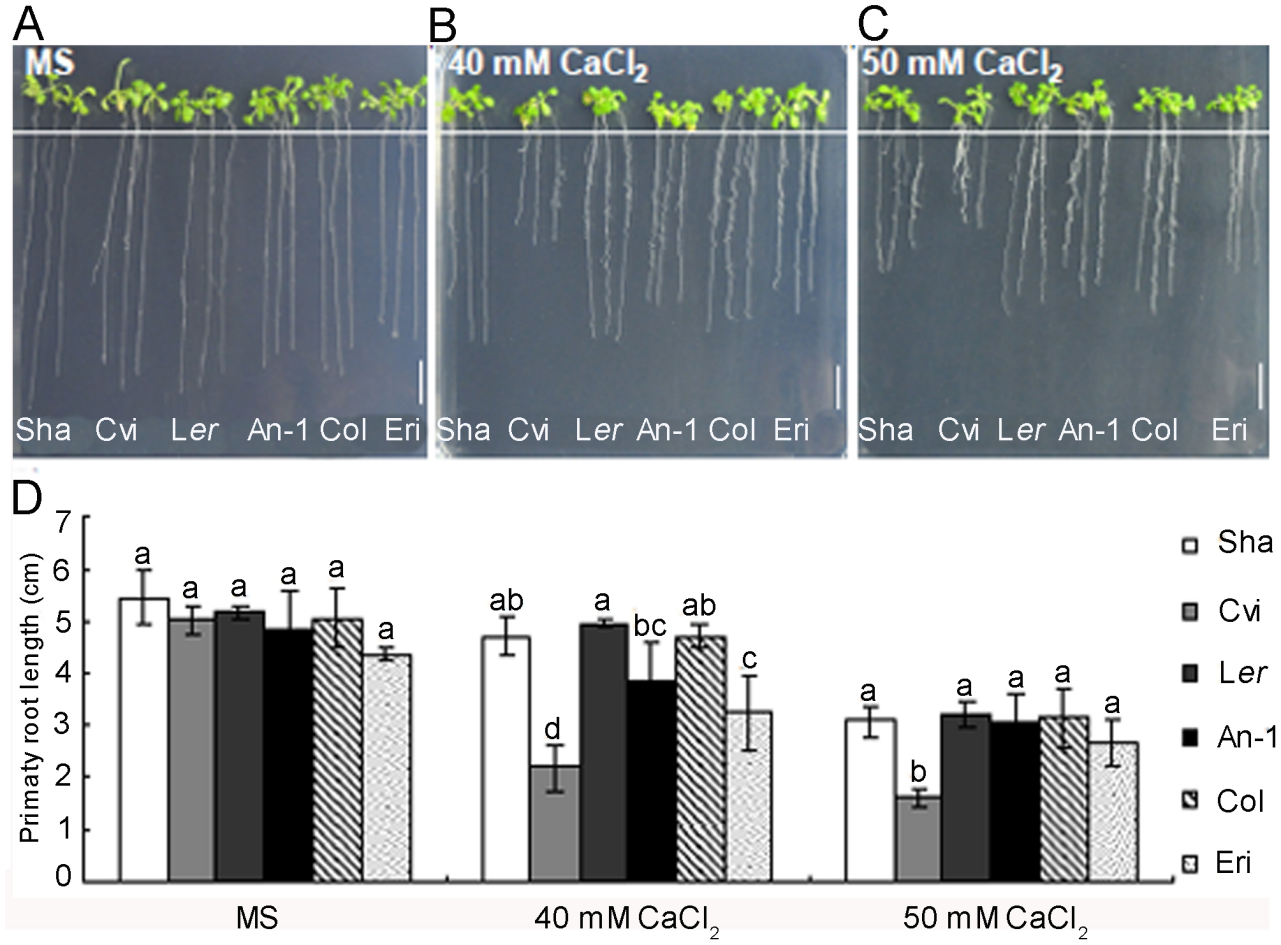
Natural variation of high Ca response in *Arabidopsis*. (A–C) Pictures of different *Arabidopsis* accessions grown on medium with or without 40 and 50 mM CaCl_2_ (Scale bar = 1 cm). Shakdara (Sha, N929), Cape Verde Islands (Cvi, N902), Landsberg *erecta* (L*er,* N1642), An-1(N99), Columbia (Col-0, N1092), Eri. (D) Analysis of primary root length of *Arabidopsis* accessions under different calcium conditions. Data presented as means± SD (n≥3). The different lowercase letters on the bars indicate significantly different means (P<0.05).

In order to gain insight into the *Arabidopsis* response to high calcium stress, we compared six commonly used accessions grown under control, 40 and 50 mM CaCl_2_ conditions. Typical seedlings after treating 10 days are shown in Figure 1. Under the normal condition, no significant difference between the six *Arabidopsis* accessions was observed. In comparison with the control, the plants growing on high calcium medium showed a reduction of primary root length (Figure 1). The response was widely different among the six accessions. The primary root length of Cvi was dramatically reduced 56.4% and 67.9% under 40 and 50 mM CaCl_2_ conditions, while the primary root length of L*er* was hardly affected.

In high calcium growth conditions, L*er* always exhibited the longest primary root length and Cvi was the accessions showing the least primary root length (Figure 1D). These contrasted growth habits between L*er* and Cvi, especially under 40 mM CaCl_2_ condition, led us to investigate the molecular and genetic basis behind this natural variation.

### Ca^2+^, rather Cl^−^, largely affected primary root growth

**Figure 2:**
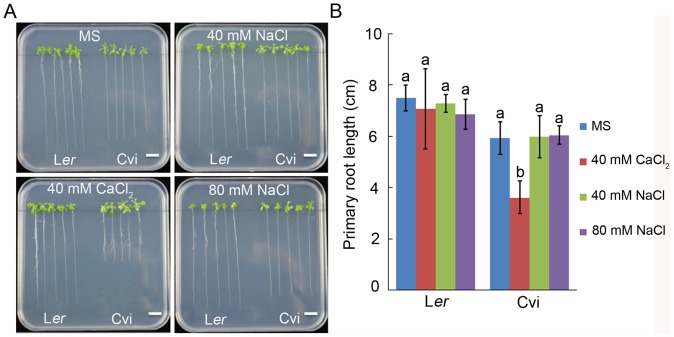
The primary root length of L*er* and Cvi seedlings on different medium with high CaCl_2,_ NaCl and KCl. (A) Pictures of L*er* and Cvi seedlings grown on different medium (Scale bar = 1 cm). (B) Primary root length analysis of L*er* and Cvi seedlings grown under different conditions. Average values ± SD are shown (n = 5). The different lowercase letters on the bars indicate significantly different means (P<0.05).

40 mM CaCl_2_ can greatly lead to reduced primary root growth of Cvi. In order to determine which was the main element (Ca^2+^ or Cl^−^) restricting the root development of *Arabidopsis*, we studied the responses of L*er* and Cvi to the salt solution containing the same amount of Cl^−^ as CaCl_2_ solution (Figure 2). No obvious reduction of the primary root length was observed under high NaCl and KCl treatments for L*er* and Cvi. But in the CaCl_2_ condition, the primary root length of Cvi was significantly reduced 39% while that of L*er* was nearly not affected with the reduction of only 5.79%. In addition, the primary root length of Cvi was about one-third of that of L*er* in 40 mM Ca(NO_3_)_2_ treatment, meanwhile the two accessions showed nearly the same primary root length on MS medium (Figure S1). By comparing the reaction of the two *Arabidopsis* accessions to different salt solutions, we could draw the conclusion that Ca^2+^ rather than Cl^−^ largely restricted the primary root length.

### Identification of QTLs involved in response to high calcium stress

**Figure 3:**
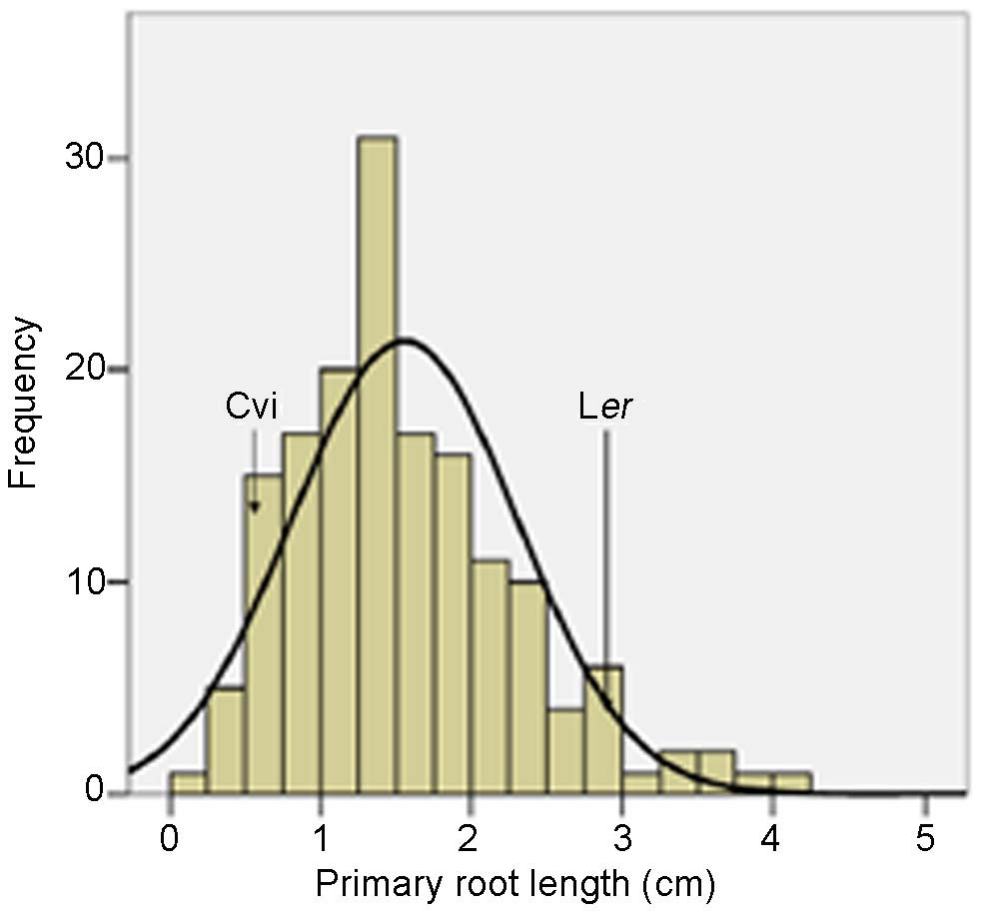
Frequency distribution of primary root length of L*er*/Cvi RIL population grown under 40 mM CaCl_2_ condition. Arrows indicate the levels in the parental lines: L*er* and Cvi.

To identify the genetic loci involved in high calcium stress response in *Arabidopsis*, the primary root length of 161 L*er*×Cvi recombinant inbred lines (RILs) was determined under control and 40 mM CaCl_2_ conditions after 10 days growth. No significant difference in the primary root length of RILs under control condition was observed. A normal distribution of primary root length under 40 mM CaCl_2_ treatment was detected, indicating that multiple genetic factors are segregated among the RIL population (Figure 3). In addition, the frequency distributions of primary root length show minor transgression in both directions. This suggests that both accessions carry genes with alleles contributing to increase or decrease the primary root length under high calcium condition.

**Figure 4:**
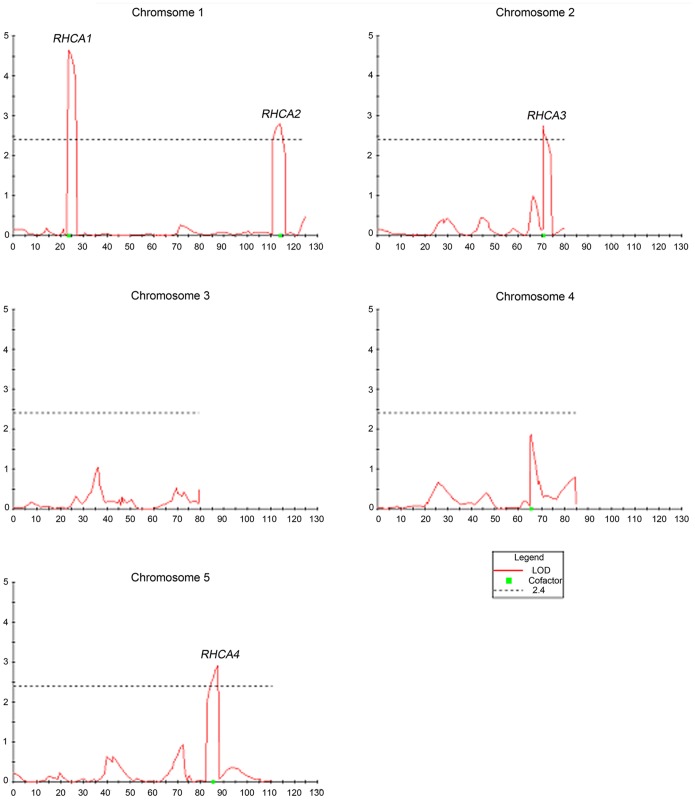
QTL analysis of L*er/*Cvi RIL population for primary root length under 40 mM CaCl_2_ condition. LOD score values from multiple-QTL model mapping (MQM) analysis of traits plotted against a linear representation of the *Arabidopsis* genome. Red lines represent LOD score values. Dashed lines indicated the threshold value. Four QTLs were identified in chrom.1, 2, and 5 and named response to high calcium (*RHCA*) 1–4.

By combining primary root length data with molecular marker data, QTL mapping was performed for primary root length response to high calcium stress. Four significant QTLs controlling primary root length under high calcium condition were detected on chromosome 1, 2 and 5 respectively (Figure 4), and named *RHCA1* and *RHCA2* (in chrom.1), *RHCA3* (chrom.2) and *RHCA4* (chrom.5), explaining total 36.8% of the variation. All the Cvi alleles of the four QTLs contributed to shorter primary root length and the L*er* alleles of the four QTLs led to longer primary root length in high Ca treatment. Epistatic interactions among QTL loci were not detected. In addition, the same QTL locations were also detected by performing QTL analysis for the ratio of primary root length of RILs in high Ca treatment to the primary root length in the control condition, confirming the existence of the four QTLs.

### Confirmation of QTL by analysis of near-isogenic lines

**Figure 5:**
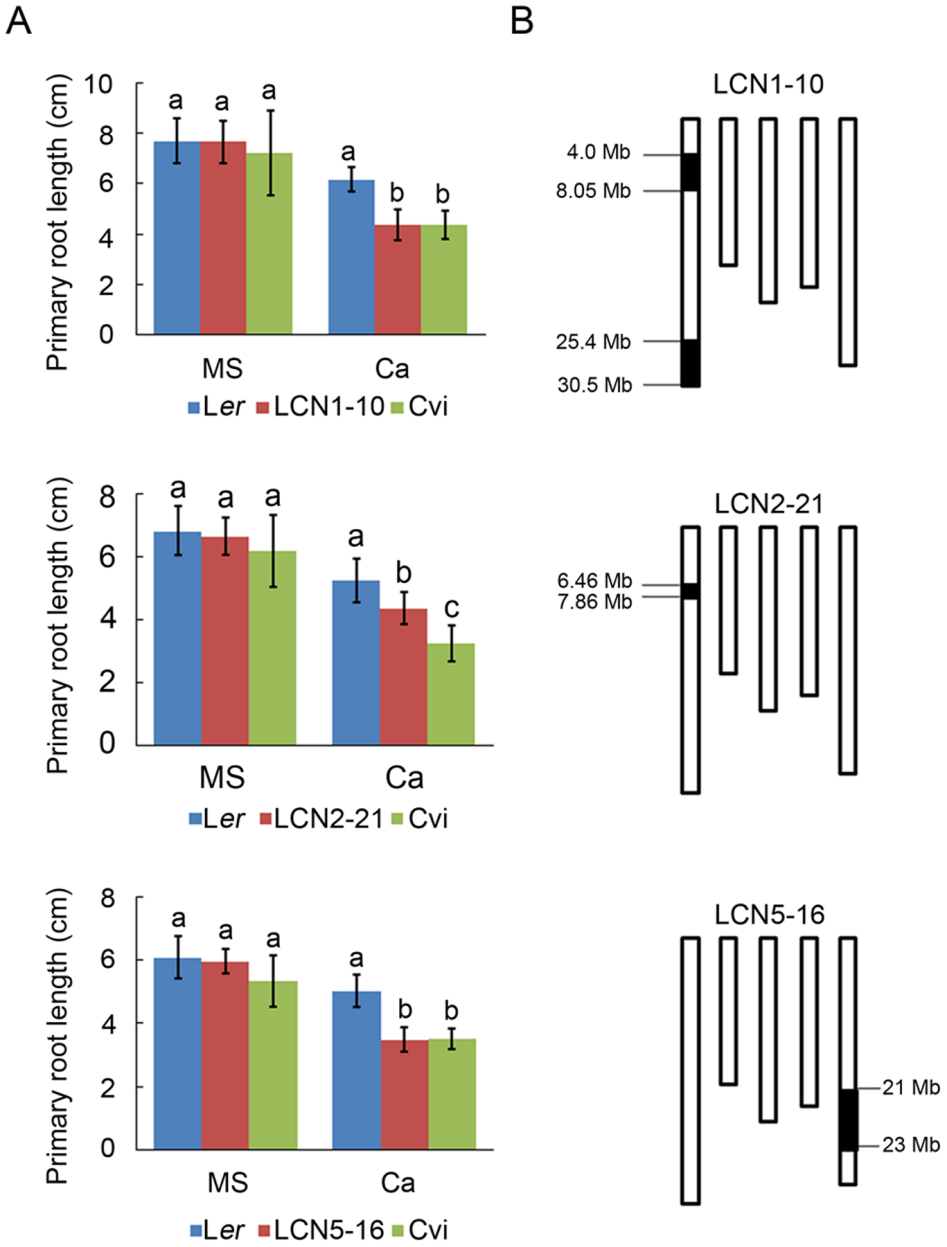
The response of L*er*/Cvi near-isogenic lines (NILs) containing QTLs on chromosome 1 and 5 to high calcium. (A) Analysis of primary root length of L*er*/Cvi NIL lines grown on different medium. Data presented as means ± SD (n≥5). The different lowercase letters on the bars indicate significantly different means (P<0.05). (B) The graphic genotypes of NIL lines. White column indicates L*er* genome, and black column indicates Cvi introgression fragment.

To validate the presence and the effect of QTLs, the primary root length was evaluated using 92 L*er*×Cvi near-isogenic lines (NILs) carrying specific Cvi introgression fragments in a L*er* genetic background [25]. NIL lines seedlings were treated with normal and 40 mM CaCl_2_ conditions for 10 days. Three NIL lines were obtained based on shorter primary root lengths than L*er* under high Ca condition (Figure 5A). Two of the three NIL lines were LCN1-10 and 2–21 carrying Cvi introgression fragments on chromosome 1. Correspondingly, the two QTLs on chromosome 1 were located at 6.46–7.86 Mb (*RHCA1*) and 25.40–30.5 Mb (*RHCA2*) respectively (Figure 5B). LCN1-10 line may contain *RHCA1* and *RHCA2*, LCN2-21 only harbors *RHCA1*. The third high Ca sensitive line was LCN5-16 carrying Cvi introgression fragment on chromosome 5, and the QTL *RHCA4* was positioned at 21–23 Mb (Figure 5B) which was consistent with the Cvi introgression fragment.

### Expression analysis of the key genes involved in response to high Ca stress in *Ler* and NILs

**Figure 6:**
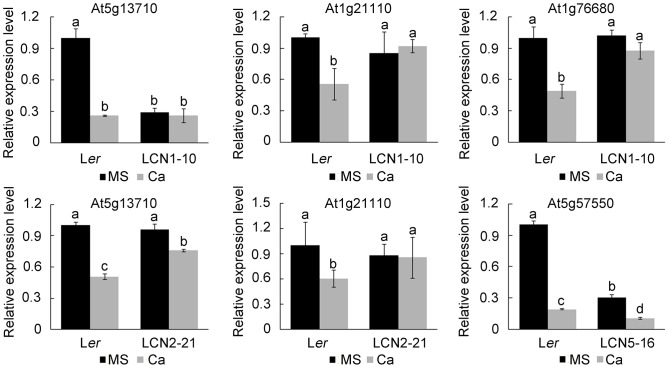
Real-time PCR analysis of genes showing different expression pattern between L*er* and NIL lines under normal and 40 mM CaCl_2_ conditions. Transcript levels of At5g13710, At1g21110, At1g76680 and At5g57550 were determined by real-time PCR. Total RNA was isolated from 5-day-old seedlings soaked in MS or MS with 40 mM CaCl_2_ solutions for 24 h. The expression values of the individual genes were normalized using the expression level of *ACTIN2* as an internal standard. The mean expression values and SD values were calculated from the results of three independent experiments. The different lowercase letters on the bars indicate significantly different means (P<0.05) for expression level.

In order to investigate the molecular basis underlying the nature variation for primary root growth in response to high Ca, we studied some known genes related to high Ca stress response including 18 genes that were up or down regulated in *Wassilewskija* (Ws) after treated with 20 mM CaCl_2_ [19] and At5g13710 (*SMT1*) and At5g15410 (*CNGC2*) which play the key roles in response to high Ca stress [7], [18]. In the end, four genes, *SMT1*, At1g21110 (*IGMT3)*, At1g76680 (*OPR1*) and At5g57550 (*XHT25*) showing different expression patterns between L*er* and NIL lines were obtained (Figure 6). The expression of At1g21110 and At1g76680 were reduced in L*er* under high Ca condition, which was consistent with the results reported by Chan et al. [19]; however, the reduced expression of At5g57550 in high Ca treatment in this study was opposite to former microarray data [19], this may be due to the different background of the material.

*SMT1* expression in L*er* was significantly reduced more than 50% after treated with 40 mM CaCl_2_. Interestingly, the expression level of *SMT1* in LCN1-10 under normal condition was similar to that in L*er* in high Ca condition. *SMT1* expression level in LCN1-10 was not affected by high Ca treatment. In contrast, *SMT1* expression level in LCN2-21 was similar to L*er* on MS medium, but in high Ca condition *SMT1* level was also obviously reduced as it did in L*er*. At1g21110 showed similar expression pattern in LCN1-10 and LCN2-21. The expression level of At1g21110 in L*er* under high Ca condition was decreased to half of that in normal condition, but the levels in LCN1-10 and LCN2-21 were not reduced by high Ca treatment, which were similar to that in L*er* under normal condition. The expression level of At1g76680 in LCN1-10 was also similar to L*er* under normal condition and was not changed by high Calcium treatment, but At1g76680 expression in L*er* under high Ca treatment fell to half of that in normal condition. The expression of At5g57550 in LCN5-16 was only 30% of L*er* on MS control medium, and its expression in both L*er* and LCN5-16 were severely reduced in 40 mM CaCl_2_ treatment.

Three of the four genes were found to be located in the QTL regions. At1g21110 located at 7.3 Mb on chromosome 1 was in the QTL *RHCA*1 region 6.46–7.86 cM. At1g76680 was at 28.7 Mb on chromosome 1 and belonged to 25.4–30.5 Mb QTL *RHCA*2 region. At5g57550 was positioned at 23.3 Mb on chromosome 5 and was in the QTL *RHCA*4 region of 21–23 Mb. At5g13710 located at 4.42 Mb on chromosome 5 was not included in the QTL regions we obtained. By the data from the 1001 genome browser on the signal website (http://signal.salk.edu/atg1001/index.php), we compared the DNA sequences of At1g21110, At1g76680 and At5g57550 genes between Cvi and L*er*. At1g21110 and At5g57550 show lots of variations especially in promoter regions between L*er* and Cvi. There are 15 SNPs and 2 single base-pair deletions in promoter region of At1g21110 and 41 SNPs and 7 single base-pair deletions in promoter region of At5g57550. These differences probably affect the expression of At1g21110 and At5g57550 under different conditions and support that the two genes could be the candidates for QTLs. There are only a few SNPs in At1g76680 between L*er* and Cvi, indicating that this gene may not be the candidate for QTLs. The amino acid sequences of three proteins show no any change between L*er* and Cvi.

## Discussion

In order to detect molecular mechanisms of plant adapted to high calcium stress, six commonly used *Arabidopsis* accessions were treated with normal and high Ca concentrations in this study. The reduction of primary root length of all six accessions in high calcium treatments is consistent with the report of root length reduction of Ws under high calcium condition [6]. Substantial quantitative variation was observed for primary root length between different accessions under high calcium conditions (Figure 1). These results indicated that *Arabidopsis thaliana* has evolved some capacities to adapt to high Ca stress and the primary root length of seedlings was a suitable index for detecting different high Ca responsive mechanisms among diverse accessions.

The primary root length of seedlings from 161 RILs under high Ca treatment showed obvious normal distribution, which indicating that plant response to high calcium stress was quantitative trait (Figure 3). By QTL analysis, four QTLs associated with the primary root length of seedlings under high calcium condition were identified in chromosome 1, 2 and 5, respectively (Figure 4). Cvi alleles of the four QTLs contributed to shortening the primary root length under high calcium condition. The QTL *RHCA1* was located in the region of 6.46–7.86 cM on chromosome 1 (Figure 5) overlaps with the QTL for calcium content in seeds of L*er* and Cvi accessions [29], indicating that *RHCA1* might be related to Ca uptake. Other three QTLs were first reported by this research. The QTLs on chromosome 1 and 5 were confirmed by NIL lines (Figure 5), but the NIL lines carrying Cvi introgression fragments on chromosome 2 covering the *RHCA3* location did not show shorter primary root length than L*er* seedlings under high Ca condition (data not shown). It is probable that the high Ca sensitive function of *RHCA*3 depends on Cvi alleles at other regions that could not be detected (epistasis).

In the study, we showed that four genes had different expression pattern between L*er* and NIL lines under normal and high Ca condition by quantitative real-time PCR analysis (Figure 6). *SMT1* could enhance high Ca resistance of plants. The *smt1* mutant showed shorter primary root length even under normal condition and a longer primary root was observed when the calcium salt in medium was decreased [18]. The primary root length of LCN1-10 was similar to that of L*er* on MS medium, but was significantly shorter than that of L*er* under high Ca condition (Figure 5A). The lower expression of *SMT1* in LCN1-10 under normal condition (Figure 6) probably contributed to the shorter root length in high Ca treatment. It was probable that the low expression of *SMT1* did not affect root growth under normal condition, but in high Ca environment, the low level of SMT1 in LCN1-10 failed to maintain root elongation efficiently leading to short roots. In addition, the lower expression level of *SMT1* on MS medium was only observed in LCN1-10 rather LCN2-21, meaning the expression of *SMT1* was suppressed by Cvi allele of 25.4–30.5 Mb region on chromosome 1. At1g21110 encodes an indole glucosinolate methyltransferase (IGMT3) and was mainly expressed in roots and hypocotyls [30]. As we know, no reports about the effects of IGMTs on root elongation and high Ca stress response were found. So how the higher expression level of At1g21110 in LCN1-10 and LCN2-21 than that in L*er* in high Ca treatment (Figure 6) affected root growth (Figure 5) under high Ca condition was unclear. At1g76680 encodes a 12-oxophytodienoic acid reductase (OPR1). OPR enzymes are responsible for the last steps on the synthesis of modified lipids like jasmonic acid (JA) and similar molecules [31]. OPR1 plays important role in JA synthesis [32]. JA inhibits seedling and root growth [33], [34]. The low expression of At1g76680 in L*er* under high Ca condition probably caused reduced jasmonic acid content and alleviated the inhibition of root length by JA. Unchanged expression of At1g76680 in LCN1-10 made JA content stable, which contributed to the inhibition of root elongation caused by high Ca. Furthermore, OPR1 is a new calmodulin binding protein (CBP) in *Arabidopsis* although interaction of this protein with calmodulin (CaM) needs to be confirmed experimentally [35]. OPR1 possibly responds to high external Ca concentration through CaM and contributes to restricting root elongation under high Ca condition. At5g57550 encodes a xyloglucan transferase/hydrolase (XTH25). XTHs play important roles in cell wall loosening, synthesis and restructuring, enabling cell expansion. The transglucosylase activity of XTHs is mainly limited to the root cell elongation zone and to the initiating root hairs, suggesting an important role of XTHs in promoting root elongation and root hair formation [36]. The high expression level of XTH25 can stimulate hypocotyls elongation and root growth [37]. The low expression of At5g57550 in LCN5-16 on MS medium (Figure 6) did not affect root growth under normal condition (Figure 5). In high Ca treatment, although At5g57550 expression in L*er* and LCN5-16 were both reduced, the high background content of XTH25 in L*er* probably alleviated the restriction of root elongation caused by high Ca. The low content XTH25 in LCN5-16 was deficient to defend high Ca stress, which contributed to the short root formation. These results imply that a complex regulatory network integrating JA, CaM and sterol metabolization pathways is involved in response to high Ca stress for *Arabidopsis* primary root growth.

In conclusion, our results illustrate that substantial genetic variation exists for the primary root length under high calcium condition between *Arabidopsis* accessions. The response of *Arabidopsis* to high Ca environment was participated by multiple QTLs and genes. The molecular mechanisms of underlying the genetic variations found in this work for high Ca response should be investigated in detail by further research. Our results provide foundation and clues for future works, which will facilitate in the comprehension of high calcium response mechanisms.

## Supporting Information

Figure S1 The primary root length of L*er* and Cvi seedlings under MS and 40 mM Ca(NO_3_)_2_ conditions.
(TIF)

## References

[1] Gilliham M, Dayod M, Hocking BJ, Xu B, Conn SJ, et al (2011) Calcium delivery and storage in plant leaves: exploring the link with water flow. J Exp Bot 62: 2233–2250. 21511913 10.1093/jxb/err111

[2] Dodd AN, Kudla J, Sanders D (2010) The language of calcium signaling. Annu Rev Plant Biol 61: 593–620. 20192754 10.1146/annurev-arplant-070109-104628

[3] Dayod M, Tyerman SD, Leigh RA, Gilliham M (2010) Calcium storage in plants and the implications for calcium biofortification. Protoplasma 247: 215–231. 20658253 10.1007/s00709-010-0182-0

[4] Chen F, Liu H, Yang H, Lai S, Cheng X, et al (2011) Quality attributes and cell wall properties of strawberries (*Fragaria annanassa* Duch.) under calcium chloride treatment. Food Chem 126: 450–459.

[5] Scaife MA (1978) Calcium-related disorders in plants - a possible explanation for the effect of weather. Plant Soil 50: 723–725.

[6] Song WY, Choi KS, Alexis D, Martinoia E, Lee Y (2011) *Brassica juncea* plant cadmium resistance 1 protein (BjPCR1) facilitates the radial transport of calcium in the root. Proc Natl Acad Sci USA 108: 19808–19813. 22089235 10.1073/pnas.1104905108 PMC3241789

[7] Chan CW, Schorrak LM, Smith RK Jr, Bent AF, Sussman MR (2003) A cyclic nucleotide-gated ion channel, CNGC2, is crucial for plant development and adaptation to calcium stress. Plant Physiol 132: 728–731. 12805601 10.1104/pp.102.019216 PMC1540323

[8] White PJ, Broadley MR (2003) Calcium in plants. Ann Bot 92: 487–511. 12933363 10.1093/aob/mcg164 PMC4243668

[9] Hirschi KD (2009) Nutrient biofortification of food crops. Annu Rev Nutr 29: 401–421. 19400753 10.1146/annurev-nutr-080508-141143

[10] Cao J, Yuan D, Pan G (2003) Some soil features in karst ecosystem. Advance In Earth Sciences 18: 37–44.

[11] Falkengren-grerup U, Quist ME, Tyler G (1995) Relative importance of exchangeable and soil solution cation concentrations to the distribution of vascular plants. Environ Exp Bot 35: 9–15.

[12] Yuan D (2001) On the karst ecosystem. Acta Geol Sin-Engl 75: 336–338.

[13] Gunter CC, Palta JP (2008) Exchangeable soil calcium may not reliably predict in-season calcium requirements for enhancing potato tuber calcium concentration. Am J Potato Res 85: 324–331.

[14] Simmons KE, Kelling KA (1987) Potato responses to calcium application on several soil types. Am Potato J 64: 119–136.

[15] Yuan DX, Cai GH (1988) The science of karst environment. Chongqing, China: Chongqing Press.

[16] Day M (2011) Protection of karst landscapes in the developing world: lessons from Central America, the Caribbean, and Southeast Asia. In: Philip E. van Beynen, editor. Karst Management. Berlin, Germany: Springer Netherlands Press. 439–458.

[17] Aguilar-Hernandez HS, Santos L, Leon-Galvan F, Barrera-Pacheco A, Espitia-Rangel E, et al (2011) Identification of calcium stress induced genes in amaranth leaves through suppression subtractive hybridization. J Plant Physiol 168: 2102–2109. 21794947 10.1016/j.jplph.2011.06.006

[18] Diener AC, Li H, Zhou W, Whoriskey WJ, Nes WD, et al (2000) Sterol methyltransferase 1 controls the level of cholesterol in plants. The Plant cell 12: 853–870. 10852933 10.1105/tpc.12.6.853 PMC149089

[19] Chan CW, Wohlbach DJ, Rodesch MJ, Sussman MR (2008) Transcriptional changes in response to growth of *Arabidopsis* in high external calcium. FEBS Lett 582: 967–976. 18307990 10.1016/j.febslet.2008.02.043

[20] Ookawa T, Hobo T, Yano M, Murata K, Ando T, et al (2010) New approach for rice improvement using a pleiotropic QTL gene for lodging resistance and yield. Nat Commun 1: 132. 21119645 10.1038/ncomms1132 PMC3065348

[21] Alonso-Blanco C, Aarts MG, Bentsink L, Keurentjes JJ, Reymond M, et al (2009) What has natural variation taught us about plant development, physiology, and adaptation? The Plant cell 21: 1877–1896. 19574434 10.1105/tpc.109.068114 PMC2729614

[22] Ashikari M, Sakakibara H, Lin S, Yamamoto T, Takashi T, et al (2005) Cytokinin oxidase regulates rice grain production. Science 309: 741–745. 15976269 10.1126/science.1113373

[23] Vaughn LM, Masson PH (2011) A QTL study for regions contributing to *Arabidopsis thaliana* root skewing on tilted surfaces. G3-Genes Genom Genet 1: 105–115. 10.1534/g3.111.000331 PMC3276130 22384323

[24] Alonso-Blanco C, Peeters AJM, Koornneef M, Lister C, Dean C, et al (1998) Development of an AFLP based linkage map of L*er*, Col and Cvi *Arabidopsis thaliana* ecotypes and construction of a L*er*/Cvi recombinant inbred line population. Plant J 14(2): 259–271. 9628021 10.1046/j.1365-313x.1998.00115.x

[25] Keurentjes JJ, Bentsink L, Alonso-Blanco C, Hanhart CJ, Blankestijn-De Vries H, et al (2007) Development of a near-isogenic line population of *Arabidopsis thaliana* and comparison of mapping power with a recombinant inbred line population. Genetics 175: 891–905. 17179089 10.1534/genetics.106.066423 PMC1800614

[26] Alonso-Blanco C, Bentsink L, Hanhart CJ, Blankestijn-de Vries H, Koornneef M (2003) Analysis of natural allelic variation at seed dormancy loci of *Arabidopsis thaliana* . Genetics 164: 711–729. 12807791 10.1093/genetics/164.2.711 PMC1462601

[27] Huang X, Schmitt J, Dorn L, Griffith C, Effgen S, et al (2010) The earliest stages of adaptation in an experimental plant population: strong selection on QTLS for seed dormancy. Mol Ecol 19: 1335–1351. 20149097 10.1111/j.1365-294X.2010.04557.x

[28] Livak KJ, Schmittgen TD (2001) Analysis of relative gene expression data using real-time quantitative PCR and the 2^−ΔΔ*C*^ _T_ method. Methods 25: 402–408. 11846609 10.1006/meth.2001.1262

[29] Vreugdenhil D, Aarts MGM, Koornneef M, Nelissen H, Ernst WHO (2004) Natural variation and QTL analysis for cationic mineral content in seeds of *Arabidopsis thaliana* . Plant Cell Environ 27: 828–839.

[30] Pfalz M, Mikkelsen MD, Bednarek P, Olsen CE, Halkier BA, et al (2011) Metabolic engineering in *Nicotiana benthamiana* reveals key enzyme functions in *Arabidopsis* indole glucosinolate modification. The Plant cell 23: 716–729. 21317374 10.1105/tpc.110.081711 PMC3077789

[31] Blanco F, Garreton V, Frey N, Dominguez C, Perez-Acle T, et al (2005) Identification of NPR1-dependent and independent genes early induced by salicylic acid treatment in *Arabidopsis* . Plant Mol Biol 59: 927–944. 16307367 10.1007/s11103-005-2227-x

[32] Kusnierczyk A, Winge P, Midelfart H, Armbruster WS, Rossiter JT, et al (2007) Transcriptional responses of *Arabidopsis thaliana* ecotypes with different glucosinolate profiles after attack by polyphagous *Myzus persicae* and oligophagous *Brevicoryne brassicae* . J Exp Bot 58: 2537–2552. 17545220 10.1093/jxb/erm043

[33] Berger S, Bell E, Mullet JE (1996) Two methyl jasmonate-insensitive mutants show altered expression of *AtVsp* in response to methyl jasmonate and wounding. Plant Physiol 111: 525–531. 12226307 10.1104/pp.111.2.525 PMC157863

[34] Creelman RA, Mullet JE (1995) Jasmonic acid distribution and action in plants: regulation during development and response to biotic and abiotic stress. Proc Natl Acad Sci USA 92: 4114–4119. 11607536 10.1073/pnas.92.10.4114 PMC41895

[35] Reddy VS, Ali GS, Reddy AS (2002) Genes encoding calmodulin-binding proteins in the *Arabidopsis* genome. J Biol Chem 277: 9840–9852. 11782485 10.1074/jbc.M111626200

[36] Rose JKC, Braam J, Fry SC, Nishitani K (2002) The XTH family of enzymes involved in xyloglucan endotransglucosylation and endohydrolysis: current perspectives and a new unifying nomenclature. Plant Cell Physiol 43: 1421–1435. 12514239 10.1093/pcp/pcf171

[37] Keuskamp DH, Sasidharan R, Vos I, Peeters AJ, Voesenek LA, et al (2011) Blue-light-mediated shade avoidance requires combined auxin and brassinosteroid action in *Arabidopsis* seedlings. Plant J 67: 208–217. 21457374 10.1111/j.1365-313X.2011.04597.x

