# Supplementary figures and images for: Identification of Quantitative Trait Loci Controlling High Calcium Response in Arabidopsis thaliana
Source: PLoS One. 2014 Nov 17;9(11):e112511. doi: 10.1371/journal.pone.0112511 (PMC4234421; doi:10.1371/journal.pone.0112511)

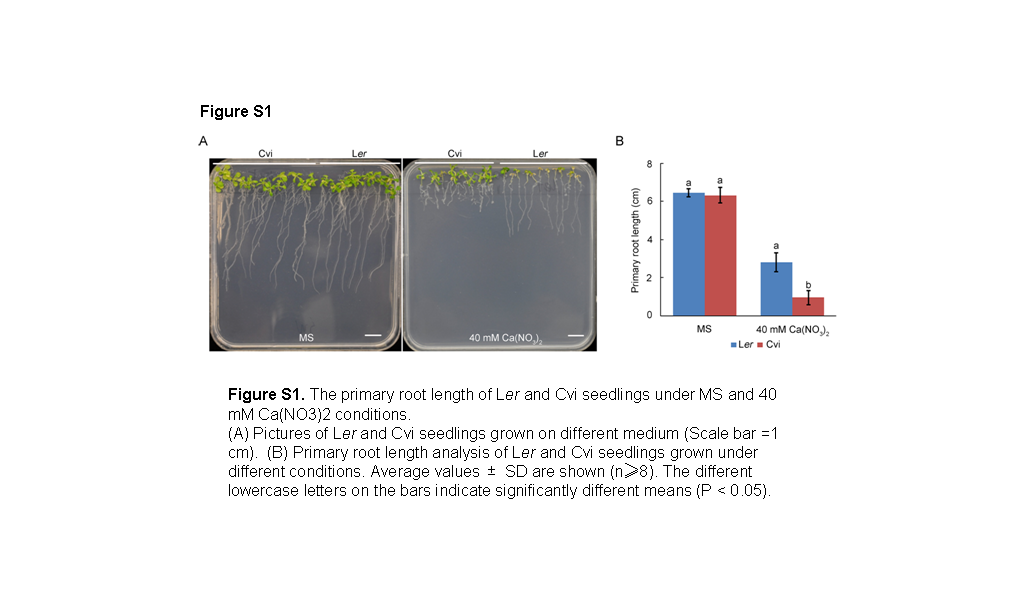

Supplement: Figure S1 — The primary root length of Ler and Cvi seedlings under MS and 40 mM Ca(NO3)2 conditions. (TIF) [file pone.0112511.s001.tif]
